# P08-04 A protocol for the development of a systems map to identify the factors that influence walking in Cork, Ireland

**DOI:** 10.1093/eurpub/ckac095.117

**Published:** 2022-08-29

**Authors:** Dylan Power, Niamh Murphy, Barry Lambe

**Affiliations:** Department of Sport and Exercise Science, Waterford Institute of Technology, Waterford, Ireland

**Keywords:** Systems, Walking, Interdisciplinary, Determinants of walking, Systems map

## Abstract

**Background:**

Systems approaches have been used in recent years in addressing complex public health problems such as obesity and physical inactivity. Depicting these ‘wicked' problems through systems maps has helped system actors better understand the entire systems in which these problems exist. Like physical activity (PA) promotion, there are multiple sectors and organisations who have a stake in the promotion of walking. However, their efforts are seldom combined, and stakeholders often work in conceptual silos when tackling the same problem. Walking has been described as a ‘best buy' for public health (Bull and Hardman, 2018). However, walking promotion requires efforts across the entire ecosystem if population-level PA goals are to be reached. The purpose of this study is to generate a systems map of the factors that influence walking in Cork.

**Methods:**

Participants of the workshops all work in Cork, Ireland. Participants remotely attended two online Zoom workshops to develop the systems map. The Australian Systems Map for Physical Activity (Bellew et al., 2020) was used as a framework in the development of the map. Semi-structured interviews (n = 5) were used to supplement the online workshops. A third online workshop is planned for October 2020 to discuss identified interventions.

**Outcomes:**

The systems map provided stakeholders with a new perspective on the complexities of the system and provided a platform to network with organisations outside of their sectors. Semi-structured interviews helped identify barriers and facilitators to working collaboratively and explored the political and commercial environment of the system. Furthermore, 19 potential interventions were identified from the workshop discussions. A selection of these will be discussed in detail in a third workshop in October 2020.

**Conclusions:**

The systems map alone will not increase walking levels across the entire system. However, it will provide stakeholders with a common visual language of the structure of the system. Thus, enabling them to identify where they sit within the system and potential leverage points they can influence. Furthermore, understanding the entire ecosystem of walking in Cork through a systems map may prove useful in other contexts when approaching the complex problem of population-level walking promotion.

